# In-vitro screening of compatible synbiotics and (introducing) “prophybiotics” as a tool to improve gut health

**DOI:** 10.1007/s10123-023-00417-2

**Published:** 2023-08-22

**Authors:** Ramesha N. Wishna-Kadawarage, Martin Jensen, Szymon Powałowski, Rita M. Hickey, Maria Siwek

**Affiliations:** 1 Department of Animal Biotechology and Genetics, Faculty of Animal Breeding and Biology, Bydgoszcz University of Science and Technology, Mazowiecka 28, 85-084 Bydgoszcz, Poland; 2 Department of Food Science, Aarhus University, AgroFoodPark 48, 8200 Århus N, Denmark; 3 Univeristy of Humanities Król Stanisław Leszczyński, Królowej Jadwigi 10, 64-100 Leszno, Poland; 4 Teagasc Food Research Centre, Moorepark, Fermoy, P61 C996 Co. Cork Ireland

**Keywords:** Antimicrobial, Gompertz model, Growth kinetics, Plant extracts, Prebiotics, Probiotics

## Abstract

Synbiotics have been intensively studied recently to improve gut health of humans and animals. The success of synergistic synbiotics depends on the compatibility of the prebiotic and probiotic components. Certain plant extracts possess both antimicrobial and prebiotic properties representing a potential use in combination with probiotics to improve the gut health. Here, we coined the term “prophybiotics” to describe this combined bioactivity. The current study aimed to select prebiotics that are preferred as an energy source and antimicrobial plant extracts which do not inhibit the growth, of six strains of lactic acid bacteria (LAB namely; *Lactiplantibacillus plantarum*, *Lacticaseibacillus casei*, *Limosilactobacillus reuteri*, *Lacticaseibacillus rhamnosus*, *Leuconostoc mesenteroides*, and *Pediococcus pentosaceus*) in-vitro to identify compatible combinations for potential synbiotic/prophybiotic use, respectively. Their growth kinetics were profiled in the presence of prebiotics: Inulin, Raffinose, and Saccharicterpenin with glucose, as the control, using carbohydrate free MRS broth media. Similarly, their growth kinetics in MRS broth supplemented with turmeric, green tea, and garlic extracts at varying concentrations were profiled. The results revealed the most compatible pairs of prebiotics and LAB. Turmeric and garlic had very little inhibitory effect on the growth of the LAB while green tea inhibited the growth of all LAB in a dose-dependent manner. Therefore, we conclude that turmeric and garlic have broad potential for use in prophybiotics, while the prebiotics studied here have limited use in synbiotics, with these LAB.

## Introduction

A healthy gut microbiome is largely responsible for maintaining innate immunity, gut barrier functioning as well as direct and indirect exclusion of pathogens (Diaz Carrasco et al. 2019). The use of probiotics, prebiotics, and synbiotics (prebiotics + probiotics) to improve gut health of humans and animal species has been studied and reported intensively in literature (as reviewed by Yadav et al. 2022). Lactic acid bacteria (LAB) have been intensively studied and are widely used as probiotics with a wide range of beneficial properties (Ljungh and Wadström 2006). Indeed, many species of LAB are listed in the updated list of qualified presumption of safety (QPS) recommended microorganisms by European Food Safety Authority (EFSA Biohaz Panel (EFSA Panel on Biological Hazards) et al. 2023) indicating the potential use of LAB in humans and animals safely.

According to the latest consensus statement by international scientific association for probiotics and prebiotics (ISAPP), based on the components and their functional role, synbiotics are divided into two main categories namely: complementary and synergistic (Swanson et al. 2020). A complementary synbiotic is a mixture of a probiotic and prebiotic chosen to act individually to improve gut health of the host while a synergistic synbiotic is a combination of live microorganisms which have beneficial effects on a host and a substrate which can selectively stimulate the growth and activity of the chosen microorganism. The selection of components in a complementary synbiotic is relatively easier given the fact that they are expected to affect the host individually. However, the selection of a components for a synergistic synbiotic requires more carefully planned studies to select the most compatible prebiotic that effectively improves the growth and functioning of the probiotic of choice (Quintero et al. 2022). Therefore, careful screening of components in a synbiotic development is crucial for its successful application. Thus, the first objective of the current study was to determine the effect of commercial oligosaccharide-based prebiotics (Inulin, Raffinose, and Sachcharicter penin) on the growth of six strains of LAB to identify the best combinations for potential synbiotic use in terms of in-vitro growth.

As an innovative approach to synergistic synbiotics with oligosaccharide based prebiotics, plant-based second-generation synbiotics have been reviewed by Sharma and Padwad (2020). Here, the authors address the problems of using conventional oligosaccharides including, supporting growth of non-beneficial bacteria, inconsistent observations in clinical endpoints, and lack of inherent bioactivity for improving the gut health (Bindels et al. 2015; Krumbeck et al. 2016) and propose plant-based polyphenolic substrates as a better companion for probiotics in synergistic synbiotics. Among these plant-based bioactives, turmeric (*Curcuma longa*) (Scazzocchio et al. 2020), green tea (*Camellia sinensis*) (Jung et al. 2017), and garlic (*Allium sativum*) (Chen et al. 2020) have shown pronounced effects in modulating the gut microbiome and improving gut-associated immunity and overall gut health in many species. Moreover, the literature indicates that these plant extracts could also display prebiotic properties on LAB (Lu et al. 2021; Sunu et al. 2019; Yazdi et al. 2019). We coined the term **Prophybiotics **(probiotic + phytobiotic) to describe this approach where we aim to utilize these beneficial health effects of these phytobiotics and probiotics synergistically in improving gut health of human and animal species. However, as these plant extracts also contain antimicrobial compounds (turmeric: Adamczak et al. 2020, green tea: Gopal et al. 2016, and garlic: Bhatwalkar et al. 2021), it is important to confirm that the growth of probiotics used in combination might not be inhibited by these antimicrobial phytobiotics. Thus, the second objective of the current study was to assess the growth of six LAB in the presence of the turmeric, green tea, and garlic extracts to assess their effects on the growth.

## Materials and methods

### Probiotics, prebiotics, and plant extracts

A total of six LAB, namely, *Lactiplantibacillus plantarum* B/00166 (LP)*, Lacticaseibacillus casei* B/00164 (LC), *Limosilactobacillus reuteri* B/00281 (LR), *Lacticaseibacillus rhamnosus* B/00279 (LRh), *Leuconostoc mesenteroides* B/00288 (LM), and *Pediococcus pentosaceus* B/00165 (PP) provided by JHJ sp. z o.o., Nowa Wieś, Poland, were used. All the strains were identified using 16S rRNA sequencing and deposited at the Polish Collection of Microorganisms located in Wrocław.

Three commercial prebiotics, namely, Raffinose VWR J392 (RAF), Saccharicterpenin (SAC) (Hubei, China), and Inulin Orafti® HPX (INU) (Mannheim, Germany), were used to determine the substrate preference of the LAB. Three plant extracts, namely, turmeric, green tea, and garlic, were used in the current study to determine their effects on probiotic growth. Green tea (spray-dried aqueous extract) and turmeric (spray-dried product of alcoholic extract of turmeric rhizomes) extracts were provided by Kaesler Gmbh, Cuxhaven, Germany. Approximately 67.5% polyphenols and 0.4% caffeine were present in the green tea extract while 2% curcumin was present in the turmeric extract used in the current study. The garlic (cultivar: Thermodrome) used in experiment was organically grown in the 2021 season in Aarhus University, Department of Food Science at Research Centre at Årslev, Funen, Denmark.

### Pre-handling of bacterial strains

All strains were retrieved from the stock cultures stored at −80°C. A loop of stock cultures were streaked on MRS agar (Merck 1.10660, Germany) plates and incubated at 37°C for 48 h to obtain isolated single colonies. A single colony was then inoculated in 10 ml of MRS broth (Merck 1.10661, Germany) and incubated for 24 h at 37°C. Two steps of subculturing were performed transferring 100 µl of overnight cultures to 10 ml of MRS broth to regain the viability after long-term storage at −80°C, and 1 ml of the second subculture incubated for 20 h was centrifuged at 13,000 rpm for 20 min to remove spent media. The cell pellet was re-suspended in 1 ml of ringer’s solution (Merck 1.15525, Germany) to prepare the inoculum for the experiment.

### Preparation of media for prebiotic assays

In order to determine the substrate preference of different LAB, a carbohydrate-free MRS (cfMRS) broth was prepared according to the formula listed in Table 1, from first principles. RAF, SAC, and INU were supplemented to the cfMRS medium separately, at 18 g/l concentration. The same concentration of D + glucose (Merck G8270) was supplemented as the control of the prebiotic assays. Each supplemented medium was then filter sterilized using 0.2-µm syringe filters (Merck WHA69012502).

**Table 1:** Formula of cfMRS preparation for prebiotic assays

Ingredient	Amount per liter
Oxoid peptone	10 g
Yeast extract	5 g
Tween 80	1 ml
K_2_HPO_4_	2 g
Sodium acetate	5 g
Triammonium citrate	2 g
MgSO_4_·7H_2_O	0.2 g
MnSO_4_·4H_2_O	0.05 g

### Preparation of plant extracts for growth curve analysis

#### Turmeric and green tea extracts (CUR and GT)

Turmeric and green tea extracts (spray-dried products in fine powder form) were measured in required quantities and directly dissolved in MRS broth at respective concentrations of supplementation. Finally, the supplemented broth media were filter sterilized using 0.2 µm syringe filters.

#### Garlic extract (G)

Fresh garlic bulbs cv. Thermodrome were chopped in to 3–5 mm slices and air-dried for 2 days at 40°C and 5 days at 50°C. Then, air-dried garlic chips were milled into powder and subsequently sieved with a 1 mm sieve. This powder was stored at −20 °C until usage. Of sieved garlic powder, 1.25 g was incubated with 10 ml of distilled water at room temperature to activate the alliinase enzyme reaction to produce allicin from alliin. First, the mixture was mixed using a vortex mixer briefly for 20 s and then shaken for 8 min at 550 rpm. After, the mixture was left for sedimentation for another 2 min. Finally, the mixture was centrifuged at 10,000 rpm for 5 min, and the supernatant was obtained. This was filter sterilized using a 0.2 µm syringe filter before it was added to MRS broth for the growth kinetic assays.

### Experimental design

Growth kinetic assays for the six LAB with respective prebiotic and plant extract supplementation (Table 2) were performed in 96-well plates (TPP B-0683). Plates were incubated at 37°C for 30 h under aerobic conditions in Hidex Sense microplate reader, and absorbance at an optical density 600 (OD600) was measured at hourly intervals. Plates were shaken orbitally at 300 rpm speed for 10 s before taking each reading. For each treatment, a negative control (without bacteria) was used as a blank. The average absorbance from triplicate wells/LAB/treatment was used to graph the growth curves using GraphPad Prism 9.5.0.

**Table 2:** Prebiotic and plant extract supplements used in the experiment

Treatment		Annotation	Concentration (w/v)
Prebiotics	Raffinose	RAF	1.8%
	Saccharicterpenin	SAC	
	Inulin	INU	
Plant extracts	Turmeric	CUR1	0.06%
		CUR2	0.1%
		CUR3	0.6%
	Green tea	GT1	0.06%
		GT2	0.1%
		GT3	0.6%
	Garlic	G1	0.25%
		G2	0.5%
		G3	1%

### Data illustration and statistical analysis

The average values of triplicate growth curves were plotted using GraphPad Prism 9.0 version. The growth curve data of the triplicates was applied in to the Gompertz model using “nls” function in R software 4.3.1 version to obtain maximum OD/growth, maximum growth rate, and lag time. The growth parameters extracted from triplicate growth curves of different levels of each supplement were compared using one-way ANOVA test in R software 4.3.1 version. The mean comparison was performed using the Tukey’s honest significant difference (HSD) test.

## Results

### Growth of lactic acid bacteria strains supplemented with different energy sources

**Fig. 1:**
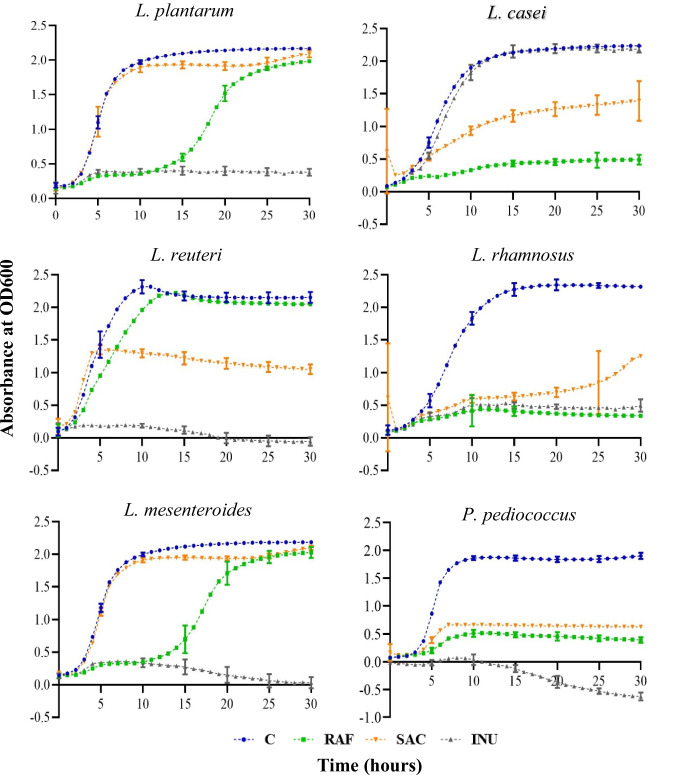
Growth kinetics (OD600 absorbance vs time (h)) for six LAB in the cfMRS media supplemented with different commercial prebiotics (1.8% w/v). C: control supplemented with glucose is indicated in blue color. RAF: supplemented with Raffinose is indicated in green color. SAC: supplemented with Saccaricterpenin is indicated in orange color. INU: supplemented with Inulin is indicated in ash color (error bars: 95% confidence interval)

The growth of the six LAB strains when supplemented with different commercial prebiotics as the sole energy source is shown in Fig. 1 and Table 3. The growth data of *L. casei*, *L. rhamnosus*, and *P. pentosaceus* when supplemented with Raffinose did not fit the standard Gompertz model where they displayed poor growth as compared to the control group (− 80%, − 82%, and − 72%, respectively). This indicates that these LAB strains did not prefer RAF as their energy source. Nevertheless, the remaining LAB strains (*L. plantarum*, *L. reuteri*, and *L. mesenteroides*) displayed a considerable growth when supplemented with Raffinose although the maximum growth/OD and maximum growth rate were lower and lag time was higher than the respective values of the control.

**Table 3:** The growth parameters (mean ± SD) of lactic acid bacteria (LAB) when different carbohydrate sources were used (C: glucose/RAF: Raffinose/INU: Inulin and SAC: Saccharicterpenin) as the sole energy source

LAB	C	RAF	INU	SAC	Significance
	Max OD/growth				
*L. plantarum*	2.13 ± 0.01^a^	2.03 ± 0.01^b^	NA	1.95 ± 0.02^c^	***
*L. casei*	2.21 ± 0.01^a^	NA	2.19 ± 0.03^a^	1.36 ± 0.09^b^	***
*L. reuteri*	2.12 ± 0.03^a^	2.11 ± 0.00^b^	NA	NA	*
*L. mesenteroides*	2.15 ± 0.01^a^	2.05 ± 0.03^b^	NA	1.97 ± 0.01^a^	***
	Max growth rate				
*L. plantarum*	1.07 ± 0.03^b^	0.52 ± 0.01^c^	NA	1.14 ± 0.02^a^	***
*L. casei*	0.80 ± 0.01^a^	NA	0.83 ± 0.04^a^	0.22 ± 0.01^b^	***
*L. reuteri*	1.29 ± 0.06^a^	0.84 ± 0.0^b^	NA	NA	***
*L. mesenteroides*	1.07 ± 0.01^b^	0.60 ± 0.02^c^	NA	1.20 ± 0.01^a^	***
	Lag time				
*L. plantarum*	2.68 ± 0.08^b^	13.58 ± 0.27^a^	NA	2.75 ± 0.12^b^	***
*L. casei*	2.70 ± 0.14	NA	3.68 ± 0.05	2.32 ± 1.6	NS
*L. reuteri*	1.96 ± 0.04^b^	2.39 ± 0.02^a^	NA	NA	***
*L. mesenteroides*	2.41 ± 0.04^b^	12.89 ± 0.56^a^	NA	2.86 ± 0.05^b^	***

The alphabetical order of superscripts indicate the statistically different (Tukey HSD) means of treatment levels in descending order. “NA” indicates the treatments where growth of the LAB was not observed (growth data were not fitted to Gompertz model)

*NS* not significant

***p value > 0.0001, **p > 0.001, *p > 0.01

Interestingly, Inulin was efficiently utilized as an energy source by *L. casei* where all the growth parameters when supplemented with Inulin were statistically similar to the control. Surprisingly, INU was not preferred as an energy source by the remaining five LAB stains where they displayed a considerably lower growth compared to the control (*L. plantarum* − 81%, *L. reuteri* − 94%, *L. rhamnosus* − 78%, *L. mesenteroides* − 87% and *P. pentosaceus* − 100%) at 15 h of incubation.

Moreover, *L. mesenteroides* displayed statistically similar maximum growth/OD and lag time and higher maximum growth rate compared to the respective values of the control when supplemented with Saccharicterpenin. Similarly, the Saccharicterpenin-supplemented *L. plantarum* showed a higher maximum growth rate compared to that of the control although it did not reach the maximum growth/OD of the control. The remaining strains did not show promise on utilizing Saccharicterpenin as an energy source successfully.

Given these results, Saccharicterpenin with *L. plantarum* or *L. mesenteroides* and Inulin with *L. casei* can be selected as compatible pairs for potential synergistic synbiotic production. Interestingly, *L. rhamnosus* and *P. pentosaceus* strains tested in the current study did not show a compatibility with any of the prebiotics studied as potential synbiotic products.

### Growth of lactic acid bacteria strains supplemented with turmeric extract

**Fig. 2:**
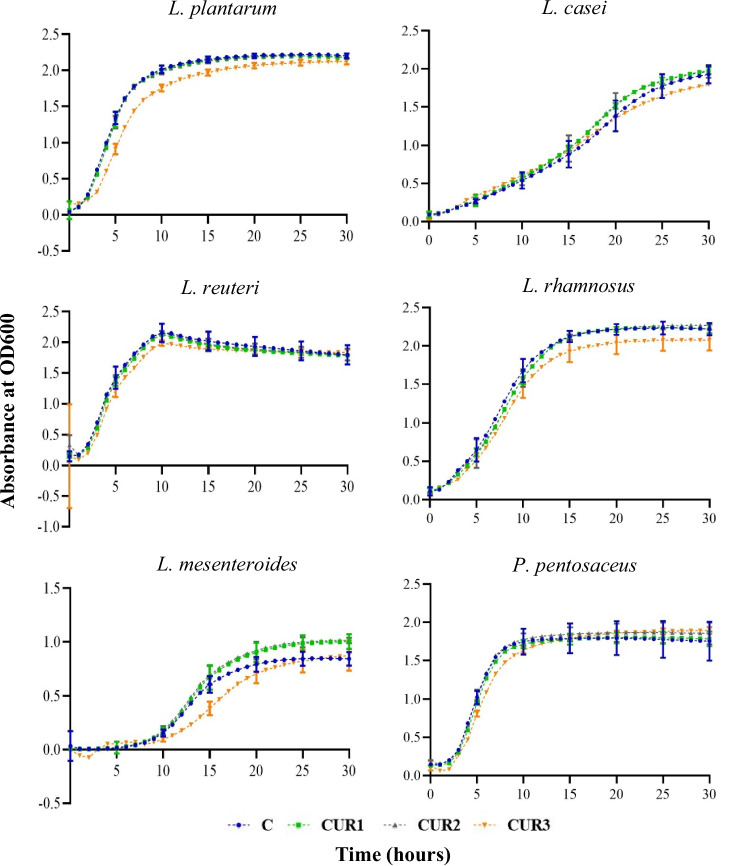
Growth kinetics (OD600 absorbance vs time (h)) for six LAB in MRS media supplemented with different concentrations of turmeric extract. C: control media without any turmeric extract supplementation is indicated in blue color. CUR1: supplemented with 0.06% (w/v) turmeric extract is indicated in green color. CUR2: supplemented with 0.1% (w/v) turmeric extract is indicated in grey color. CUR3: supplemented with 0.6% (w/v) turmeric extract is indicated in orange color. Error bars: 95% confidence interval

Growth of six LAB strains supplemented with varying concentrations of turmeric extract is shown in Table 4 and Fig. 2. Interestingly, *P. pentosaceus* and *L. mesenteroides* displayed the prebiotic effects with the turmeric extract supplementation at all three concentrations studied. *P. pentosaceus* displayed a statistically similar maximum growth/OD and maximum growth rate at all the concentrations tested compared to the control. Interestingly, it further showed a lower lag time when supplemented with the highest concentration (CUR3 0.6%) of the turmeric extract. Moreover, *L. mesenteroides* displayed higher maximum growth/OD and maximum growth rate compared to other treatment levels and the control when supplemented with the highest concentration of the turmeric extract. Furthermore, *L. reuteri* displayed a statistically similar maximum growth/OD and lag time with the supplementation of all three levels of turmeric extract although the maximum growth rate of the highest concentration was statistically lower than that of the other treatment levels and the control. All in all, the results indicate that 0.06% and 0.1% turmeric extract supplementation did not cause any inhibition (which could be expected due to the curcumin effects) to all six LAB strains studied in the current study. Therefore, according to our results, 0.1% turmeric extract can be selected as a suitable candidate for potential prophybiotic formulation in combination with all six LAB studied while with *P. pentosaceus* and *L. mesenteroides*, it could be increased up to 0.6% to maximize the benefits of the combination.

**Table 4:** The growth parameters (mean ± SD) of lactic acid bacteria (LAB) when different levels of turmeric (C: zero turmeric, control/CUR1 0.06%/CUR2 0.1% and CUR3 0.6%) were supplemented to the MRS broth media

LAB	C	CUR1	CUR2	CUR3	Significance
	Max OD/growth				
*L. plantarum*	2.19 ± 0.01^a^	2.17 ± 0.01^a^	2.17 ± 0.01^a^	2.11 ± 0.02^b^	***
*L. casei*	2.5 ± 0.04^a^	2.37 ± 0.04^b^	2.32 ± 0.02^b^	2.35 ± 0.08^b^	**
*L. reuteri*	1.96 ± 0.06	1.91 ± 0.03	1.93 ± 0.02	1.88 ± 0.01	NS
*L. rhamnosus*	2.25 ± 0.03^a^	2.26 ± 0.01^a^	2.28 ± 0.00^a^	2.08 ± 0.06^b^	***
*L. mesenteroides*	0.85 ± 0.03^b^	0.86 ± 0.00^b^	0.92 ± 0.12^ab^	1.06 ± 0.00^a^	**
*P. pentosaceus*	1.78 ± 0.09	1.77 ± 0.07	1.85 ± 0.01	1.85 ± 0.03	NS
	Max growth rate				
*L. plantarum*	0.94 ± 0.04^a^	0.97 ± 0.03^a^	0.97 ± 0.05^a^	0.59 ± 0.01^b^	***
*L. casei*	0.24 ± 0.01^b^	0.26 ± 0.01^a^	0.27 ± 0.01^a^	0.20 ± 0.01^c^	***
*L. reuteri*	0.24 ± 0.06^a^	0.26 ± 0.01^a^	0.27 ± 0.05^a^	0.20 ± 0.01^b^	**
*L. rhamnosus*	0.62 ± 0.01	0.62 ± 0.00	0.59 ± 0.03	0.64 ± 0.02	NS
*L. mesenteroides*	0.26 ± 0.01^b^	0.27 ± 0.00^ab^	0.27 ± 0.01^ab^	0.28 ± 0.00^a^	*
*P. pentosaceus*	1.04 ± 0.07	0.99 ± 0.02	1.03 ± 0.03	1.11 ± 0.61	NS
	Lag time				
*L. plantarum*	1.10 ± 0.06^b^	1.43 ± 0.09^b^	1.43 ± 0.20^b^	2.49 ± 0.20^a^	***
*L. casei*	5.76 ± 0.62^a^	5.63 ± 0.24^a^	5.92 ± 0.51^a^	1.57 ± 1.23^b^	***
*L. reuteri*	1.87 ± 0.08	2.10 ± 0.11	2.17 ± 0.39	2.10 ± 0.01	NS
*L. rhamnosus*	2.82 ± 0.37^b^	3.39 ± 0.18^b^	2.99 ± 0.7^b^	4.71 ± 0.10^a^	**
*L. mesenteroides*	8.68 ± 0.30	8.48 ± 0.00	8.51 ± 0.04	8.56 ± 0.00	NS
*P. pentosaceus*	2.65 ± 0.13^a^	2.70 ± 0.03^a^	2.67 ± 0.12^a^	2.40 ± 0.11^b^	*

The alphabetical order of superscripts indicate the statistically different (Tukey HSD) means of treatment levels in descending order

*NS* not significant

***p value > 0.0001, **p > 0.001, *p > 0.01

### Growth of lactic acid bacteria strains supplemented with green tea extract

**Fig. 3:**
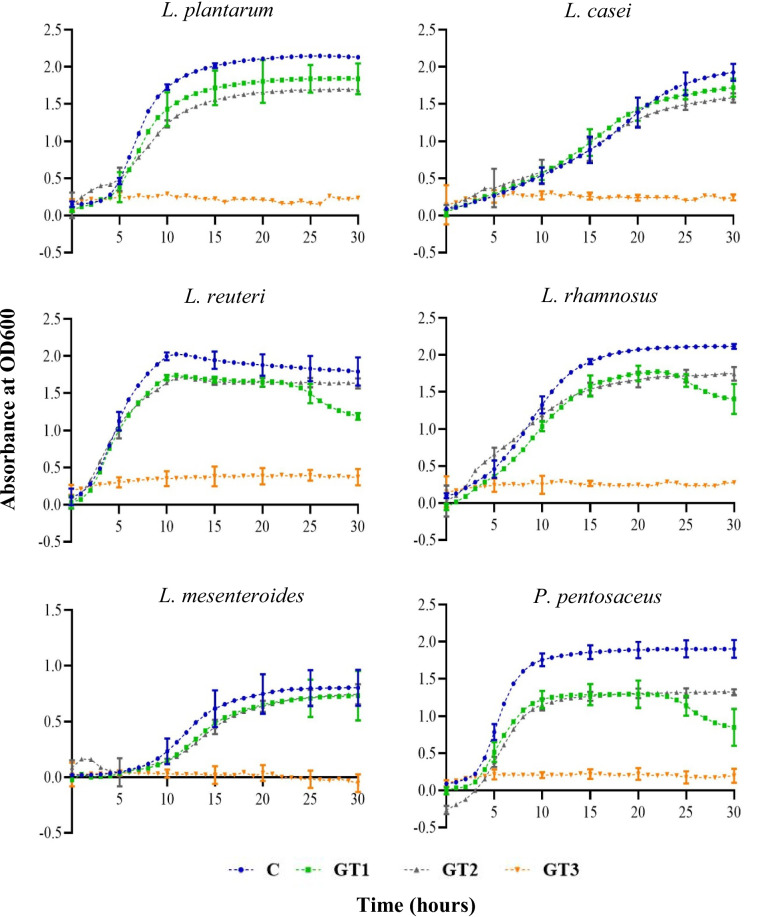
Growth kinetics (OD600 absorbance vs time (h)) for six LAB in MRS media supplemented with different concentrations of green tea extract. C: control media without any green tea extract supplementation is indicated in blue color. GT1: supplemented with 0.06% (w/v) green tea extract is indicated in green color. GT2: supplemented with 0.1% (w/v) green tea extract is indicated in grey color. GT3: supplemented with 0.6% (w/v) green tea extract is indicated in orange color. Error bars: 95% confidence interval

The growth of the six LAB strains supplemented with varying concentrations of green tea extract (Table 5 and Fig. 3) revealed that green tea displays inhibitory effect towards the growth of these LAB strains tested in the current study. At the highest concentration (GT3 0.6%), there was a strong inhibition of all six LAB studied (*L. plantarum* − 89%, *L. casei* − 70%, *L. reuteri* − 80%, *L. rhamnosus* − 86%, *L. mesenteroides* − 97%, and *P. pentosaceus* − 88% at 15 h) compared to the control growth. Thus, these growth data did not fit to the Gompertz growth model successfully. Notably, the maximum growth/OD of all LAB strains at all the levels of green tea extract supplementation was statistically lower compared to that of the respective controls except for *L. mesenteroides*. Although statistically similar maximum growth was observed at all levels of green tea extract supplementation, the growth rate of the *L. mesenteroides* was significantly lower compared to the control indicating that the maximum growth was somehow achieved at a slower pace with the green tea supplementation. Additionally, *L. casei* and *L. reuteri* displayed similar maximum growth rate as compared to the respective controls when supplemented with the two lower concentrations (0.06% and 0.1%) of green tea extract despite their lower maximum growth/OD achieved. Visually, *L. casei* displayed the highest resistance to inhibition by GT with 12% more growth observed with GT1 treatment (0.06%) compared to growth in the control media at 15 h although achieved the stationary phase at a lower OD. Given these results, green tea extract (at any concentration studied) was not selected as a suitable candidate for potential prophybiotic formulation as it showed negative effects on the growth of most of LAB strains studied.

**Table 5:** The growth parameters (mean ± SD) of lactic acid bacteria (LAB) when different levels of green tea extract (C: zero green tea, control/GT1 0.06%/GT2 0.1%) were supplemented to the MRS broth media

LAB	C	GT1	GT2	Significance
	Max OD/growth			
*L. plantarum*	2.12 ± 0.01^a^	1.82 ± 0.00^b^	1.70 ± 0.01^c^	***
*L. casei*	2.50 ± 0.04^a^	1.93 ± 0.03^b^	1.93 ± 0.09^b^	***
*L. reuteri*	1.9 ± 0.06^a^	1.57 ± 0.04^b^	1.66 ± 0.02^b^	***
*L. rhamnosus*	2.13 ± 0.01^a^	1.66 ± 0.01^c^	1.77 ± 0.03^b^	***
*L. mesenteroides*	0.76 ± 0.00	0.74 ± 0.04	0.74 ± 0.04	NS
*P. pentosaceus*	1.88 ± 0.04^a^	1.19 ± 0.03^c^	1.31 ± 0.02^b^	***
	Max growth rate			
*L. plantarum*	0.79 ± 0.01^a^	0.62 ± 0.01^b^	0.41 ± 0.03^c^	***
*L. casei*	0.24 ± 0.01^a^	0.24 ± 0.00^a^	0.18 ± 0.02^b^	**
*L. reuteri*	1.06 ± 0.06^a^	0.94 ± 0.04^a^	0.75 ± 0.03^b^	***
*L. rhamnosus*	0.54 ± 0.01^a^	0.48 ± 0.01^b^	0.37 ± 0.01^c^	***
*L. mesenteroides*	0.76 ± 0.00^a^	0.74 ± 0.00^b^	0.74 ± 0.01^b^	***
*P. pentosaceus*	0.97 ± 0.2^a^	0.78 ± 0.02^b^	0.63 ± 0.01^c^	***
	Lag time			
*L. plantarum*	3.80 ± 0.02	3.67 ± 0.21	3.10 ± 0.69	NS
*L. casei*	3.70 ± 2	4.56 ± 0.65	2.54 ± 1.75	NS
*L. reuteri*	2.31 ± 0.25^a^	2.01 ± 0.12^a^	0.93 ± 0.61^b^	*
*L. rhamnosus*	3.79 ± 0.45^a^	3.90 ± 0.71^a^	− 1.92 ± 0.20^b^	**
*L. mesenteroides*	7.62 ± 0.00^b^	7.99 ± 0.86^b^	9.65 ± 1.13^a^	*
*P. pentosaceus*	3.06 ± 0.05^b^	3.28 ± 0.10^a^	2.28 ± 0.16^c^	***

The alphabetical order of superscripts indicates the statistically different (Tukey HSD) means of treatment levels in descending order

*NS* not significant

***p value > 0.0001, **p > 0.001, *p > 0.01

### Growth of lactic acid bacteria strains supplemented with garlic extract

**Fig. 4:**
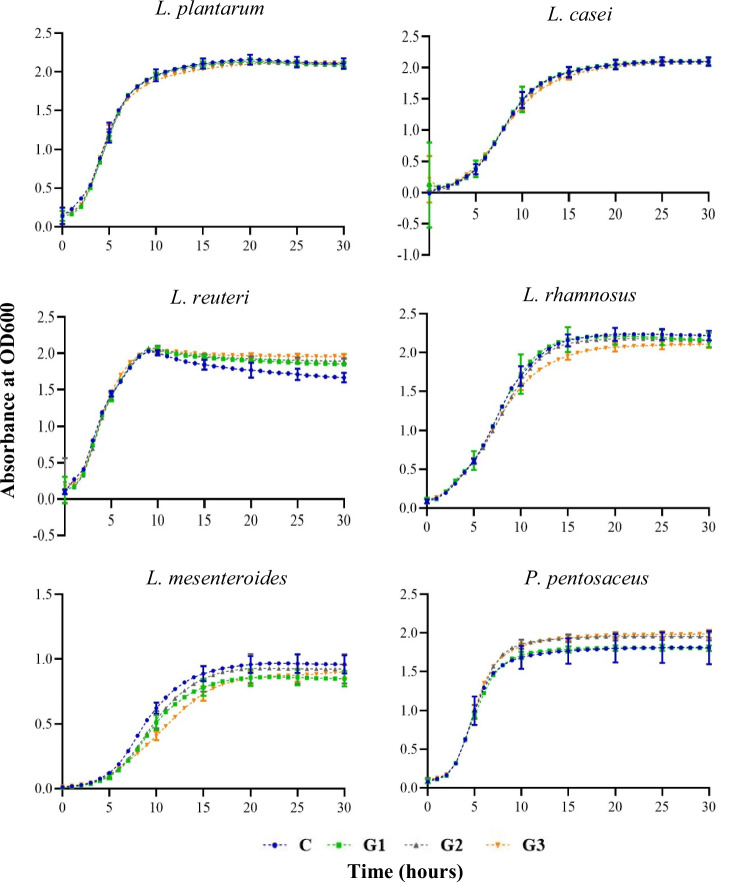
Growth kinetics (OD600 absorbance vs time (h)) for six LAB in MRS media supplemented with different concentrations of garlic extract. C: control media without any garlic extract supplementation is indicated in blue color. G1: supplemented with 0.25% (w/v) garlic extract is indicated in green color. G2: supplemented with 0.5% (w/v) garlic extract is indicated in grey color. G3: supplemented with 1% (w/v) garlic extract is indicated in orange colr. Error bars: 95% confidence interval

The growth of the six LAB strains supplemented with varying concentrations of garlic extract is indicated in Table 6 and Fig. 4. Indeed, the results of the garlic extract supplementation showed the promise for prophybiotic formulation with the LAB used in the current study. Interestingly, garlic extract supplementation displayed prebiotic effects on *L. reuteri* and *P. pentosaceus* where they display higher maximum growth with the supplementation of garlic extract as compared to the respective controls. Moreover, all the strains indicated non-inhibitory effects with the supplementation of garlic extract generally, 0.5% being the most beneficial concentration to almost all the strains studied. Therefore, 0.5% garlic extract was selected as a suitable candidate for potential prophybiotic formulation with all six LAB studied.

**Table 6:** The growth parameters (mean ± SD) of lactic acid bacteria (LAB) when different levels of garlic extract (C: zero garlic, control/G1 0.25%/G2 0.5% and G3 0.1%) were supplemented to the MRS broth media

LAB	C	G1	G2	G3	Significance
	Max OD/growth				
*L. plantarum*	2.13 ± 0.03	1.95 ± 0.28	2.10 ± 0.01	2.09 ± 0.02	NS
*L. casei*	2.09 ± 0.03	2.10 ± 0.02	2.08 ± 0.01	2.09 ± 0.02	NS
*L. reuteri*	1.81 ± 0.03^c^	1.92 ± 0.01^b^	1.95 ± 0.01^ab^	1.99 ± 0.01^a^	***
*L. rhamnosus*	2.25 ± 0.03^a^	2.22 ± 0.02^a^	2.19 ± 0.01^a^	2.11 ± 0.03^b^	***
*L. mesenteroides*	0.97 ± 0.03^a^	0.86 ± 0.02^ab^	0.94 ± 0.05^a^	0.90 ± 0.03^a^	*
*P. pentosaceus*	1.79 ± 0.07^b^	1.80 ± 0.01^b^	1.94 ± 0.02^a^	1.96 ± 0.02^a^	***
	Max growth rate				
*L. plantarum*	0.86 ± 0.01^b^	0.93 ± 0.01^a^	0.90 ± 0.01^a^	0.82 ± 0.01^c^	***
*L. casei*	0.64 ± 0.01^a^	0.65 ± 0.01^a^	0.65 ± 0.01^a^	0.55 ± 0.01^b^	***
*L. reuteri*	1.25 ± 0.04	1.21 ± 0.02	1.24 ± 0.06	1.23 ± 0.03	NS
*L. rhamnosus*	0.68 ± 0.02^a^	0.71 ± 0.00^a^	0.66 ± 0.02^a^	0.57 ± 0.04^b^	***
*L. mesenteroides*	0.31 ± 0.01^a^	0.27 ± 0.01^b^	0.29 ± 0.02^ab^	0.21 ± 0.01^c^	***
*P. pentosaceus*	0.93 ± 0.03^b^	0.90 ± 0.01^bc^	1.00 ± 0.01^a^	0.97 ± 0.01^ab^	***
	Lag time				
*L. plantarum*	1.62 ± 0.01^b^	1.91 ± 0.06^a^	1.92 ± 0.11^a^	1.45 ± 0.04^b^	***
*L. casei*	3.81 ± 0.10	4.01 ± 0.38	3.93 ± 0.06	3.59 ± 0.35	NS
*L. reuteri*	1.65 ± 0.08	1.71 ± 0.03	2.01 ± 0.29	1.86 ± 0.12	NS
*L. rhamnosus*	3.16 ± 0.06^b^	3.37 ± 0.04^a^	3.39 ± 0.09^a^	2.65 ± 0.08^c^	***
*L. mesenteroides*	4.58 ± 0.06^b^	5.08 ± 0.02^a^	5.19 ± 0.17^a^	4.89 ± 0.16^ab^	**
*P. pentosaceus*	2.37 ± 0.08^c^	2.42 ± 0.03^bc^	2.62 ± 0.05^a^	2.53 ± 0.05^ab^	**

The alphabetical order of superscripts indicates the statistically different (Tukey HSD) means of treatment levels in descending order

*NS* not significant

***p value > 0.0001, **p > 0.001, *p > 0.01

## Discussion

Initial screening of the compatibility of bioactive compounds is extremely important for the success of synergistic synbiotic production (Wu et al. 2017). The current study aided in the identification of compatible bioactive substances, either commercial prebiotics or plant extracts for use in potential formulations of synergistic synbiotics or prophybiotics, respectively, with the six LAB studied. All the LAB strains used in the current study are commercially used in probiotic products for poultry and swine, produced by JHJ sp. z o.o due to their proven beneficial characteristics in improving gut health (Jhj-lavipan-2021; Smialek et al. 2018, 2019).

Commercial prebiotics, Raffinose and Inulin used in the current study, are well known for their prebiotic potential as reviewed by Anggraeni (2022) and Teferra (2021), respectively. However, in the current study, those prebiotics were not compatible with many of the LAB such as *L. rhamosus* and *P. pentosaceus* indicating the limited use of these commercial prebiotics in synergistic synbiotic application with these strains. Saccharicterpenin is a novel feed additive derived from extracts of Theaceae plants (Liu et al. 2019) containing primarily polysaccharides and triterpinoides (Liu et al. 2016) with wide variety of benefits to livestock including antioxidant activity (Liu et al. 2019), intestinal development (Peng et al. 2011), and digestive enzyme activity (Liu et al. 2016). However, its prebiotic potential has not been studied to date. Hence, to our knowledge, our study is the first to show the potential of Saccharicterpenin as a prebiotic for lactic acid bacteria. As we observed that *L. plantarum* and *L. mesenteroides* were able to use Saccharicterpenin as an energy source, Saccharicterpenin displays the potential to be utilized for developing a potential synergistic synbiotic along with these two LAB. However, further investigation is necessary to elucidate the potential of Saccharicterpenin as a prebiotic in terms of its effects on the poultry gut microbiota.

The results of supplementation of turmeric extract suggested that turmeric extract might be a suitable candidate for potential prophybiotic formulation in combination with the LAB studied. In compliance with our results, other studies have shown that turmeric enhanced the growth of probiotics such as *L. rhamnosus* GG ATCC 53103 and *Bifidobacterium animalis* BB12 (Yazdi et al. 2019) and did not inhibit the growth of *L. acidophilus* (Ilham et al. 2018), *L. acidophilus* A001F8, *L. rhamnosus* A001G8, *L. paracasei* A002C5, *L. plantarum* A003A7, and *L. casei* A003D4 (Kim et al. 2020). In addition to that, previous literature has also shown that turmeric in combination with *Lactobacillus* probiotics resulted in enhanced antimicrobial activity (Kim et al. 2020) and anti-allergic inflammatory activity (Yazdi et al. 2020) while improving poultry production parameters (Kinati et al. 2022). These studies along with our current results indicate that turmeric may be a potential candidate to use in combination with *Lactobacillus* species without affecting bacterial growth for potential prophybiotic application.

On the other hand, existing literature has shown that green tea modulates the composition of intestinal microbiota to improve overall gut health (Chen et al. 2019), while green tea in combination with probiotics reduced the high-fat-diet-induced inflammation in mice (Axling et al. 2012) and hepatorenal syndrome in rat model (Al-Okbi et al. 2019), indicating that green tea is an excellent candidate for prophybiotic application. However, our results demonstrate that supplementation of green tea extracts at higher doses can inhibit the growth of the LAB strains used. In contrast, Story et al. (2009) found that the growth of *L. acidophilus* and *L. gasseri* was increased even at higher concentrations of green tea supplementation. Moreover, several studies have reported that the count of *Lactobacillus* starter cultures in yoghurt is increased with green tea supplementation (Lim 2017; Marhamatizadeh et al. 2013; Najgebauer-Lejko 2014). Interestingly, Janiak et al. (2018) claim that the variation of effects of green tea on probiotic growth could be due to the composition of polyphenolic compounds. The authors reported that the catechins (monomeric flavan-3-ols) help to modulate the growth of microorganisms more selectively than the polymeric fraction in green tea. Proanthocyanidins will inhibit microbial growth more generally and efficiently. Therefore, these findings highlight the importance of performing the individual growth curves for selected probiotic strains with a particular green tea extract when selecting the combinations for potential prophybiotic application as the LAB strains used in the current study showed sensitivity to green tea at higher concentrations.

Our results indicated that garlic extract did not inhibit the growth of most LAB strains while it displayed prebiotic effects on some strains. Interestingly, garlic has been reported to have prebiotic effects particularly on *Lactobacillus* species (Lu et al. 2021; Sunu et al. 2019; Sutherland et al. 2009) and *Bifidobacterium* species (Zhang et al. 2013). However, some contrasting results have also been found in the literature. Altuntas and Korukluoglu (2019) and Booyens and Thantsha (2013) observed that garlic extracts display antimicrobial effects on *L. acidophilus* and *Bifidobacterium* species. However, in the latter study, the inhibition of probiotic growth by fresh garlic extracts (crushing garlic cloves) was significantly higher than that of garlic powder extract. The authors suggest that it is possibly due to the presence of more active allinase enzymes in fresh cloves when compared with the powdered garlic which will produce more allicin (the active antimicrobial compound) during the extraction process. Since powdered garlic has been used in the current study, it is possible that the allicin content in our garlic extract was less than that of the study of Booyens and Thantsha which resulted in inhibition of probiotics. However, in the same study, it was shown that the sensitivity of different probiotics to garlic extract varied. Therefore, it is also possible that the strains that we have tested in the current study are more resistant to antimicrobial effects of garlic. Therefore, it is imperative to focus on the content of the antimicrobial compounds in the phytobiotics when screening for potential prophybiotic combinations. Therefore, growth curve analysis of probiotics in each case is required to develop successful potential prophybiotics.

It is also important to highlight that the effect of the supplementation of these prebiotics and plant extracts may be different in different strains of the same LAB species owing to wide metabolic differences within the strains of LAB species. Nonetheless, considering the results of the strains used in the current study, prophybiotic formulation seemed promising as plant extracts used in the current study did not inhibit the LAB studied in two out of three species. Therefore, it shows the potential to use a mixture of these LAB along with plant extracts (turmeric or garlic) to optimize the beneficial effects on the gut health of the host. However, as the LAB were very selective in their ability to exploit the commercial prebiotics as their energy source, use of a mixture of LAB with commercial prebiotics, as a synergistic symbiotic, might not be possible due to this selectivity. Nevertheless, the main constraint of prophybiotic formulations is the differences among different cultivars or different extraction systems in terms of bioactive composition. Therefore, we suggest that more future research is necessary to elucidate the potential of prophybiotic formulation, minimizing these constraints.

## Conclusion

Garlic and turmeric extracts displayed non-inhibitory effects for all LAB strains studied indicating their potential to use in prophybiotic formulations in the future. Nevertheless, the commercial prebiotics displayed the potential as an energy substrate limited only to particular LAB indicating a limited use of these prebiotics in synergistic symbiotic formulation with the LAB studied.

## Data Availability

The datasets generated during and/or analyzed during the current study are available from the corresponding author on reasonable request.
